# Relationship Between Negative Cognition and Poor Quality of Life and Anxiety in Adolescents: A Meta-Analysis

**DOI:** 10.31083/AP46205

**Published:** 2026-02-25

**Authors:** Şenay Kılınçel, Oğuzhan Kılınçel

**Affiliations:** ^1^Department of Child and Adolescent Psychiatry, Faculty of Medicine, Istanbul Aydın University, 34295 Istanbul, Turkey; ^2^Department of Child Development, Istanbul Gelisim University Faculty of Health Sciences, 34315 Istanbul, Turkey

**Keywords:** adolescent, anxiety, cognitive dysfunction, quality of life, meta-analysis as topic, cognitive behavioral therapy, attitude, thinking

## Abstract

**Background:**

Anxiety disorders are among the most prevalent psychiatric conditions during adolescence and are closely associated with maladaptive cognitive processes and impaired quality of life (QoL). However, the magnitude of these associations and the factors moderating them remain inconsistent across studies. This meta-analysis aimed to synthesize the available empirical evidence on the relationships between negative cognitions, QoL, and anxiety in adolescents, and to examine potential moderating variables.

**Methods:**

In accordance with the PRISMA 2020 guidelines, a systematic literature search was conducted across PubMed, Embase, PsycINFO, Web of Science, Scopus, and grey literature sources. Eligible studies included adolescents aged 10–19 years and reported correlation coefficients between negative cognitions or QoL and anxiety. A total of 42 studies (N = 27,845) were included and pooled using random-effects models, with Fisher’s z-transformed correlation coefficients as the primary effect size. Moderator analyses examined the influence of measurement instruments, sample characteristics (clinical vs. non-clinical), age, gender distribution, and study quality.

**Results:**

Across 34 studies (n = 21,006), negative cognitions showed a moderate positive association with anxiety (r = 0.41, 95% CI: 0.37–0.45, *p* < 0.001). Across 26 studies (n = 15,784), QoL demonstrated a moderate inverse association with anxiety (r = –0.36, 95% CI: –0.41 to –0.31, *p* < 0.001). Substantial heterogeneity was observed for both outcomes (I^2^ = 68% for negative cognitions and 72% for QoL). Moderator analyses revealed stronger associations in clinical samples (negative cognition–anxiety r = 0.47; QoL–anxiety r = –0.42) compared with school- or community-based samples. Gender distribution significantly moderated effect sizes, with studies including more than >60% female participants reporting stronger associations (negative cognition–anxiety r = 0.44; QoL–anxiety r = –0.39, both *p* < 0.05). Measurement instruments also influenced results: the Dysfunctional Attitude Scale yielded the strongest associations between negative cognitions and anxiety (r = 0.45, *p* < 0.001), whereas QoL–anxiety associations were most pronounced when assessed using the KIDSCREEN instrument (r = –0.39, *p* < 0.001). Age group and country income level did not significantly moderate associations, although slightly stronger correlations were observed among older adolescents (15–19 years) compared with younger adolescents. Sensitivity analyses and publication bias assessments supported the robustness of the findings.

**Conclusion:**

Negative cognitions and reduced quality of life are robustly associated with anxiety in adolescents, particularly in clinical samples and in studies with a predominance of female participants. These findings provide strong support for cognitive–behavioral models of adolescent anxiety and underscore the importance of integrating cognitive restructuring with quality-of-life–enhancing strategies in prevention and intervention programs. Future longitudinal and cross-cultural research is needed to clarify causal mechanisms and to optimize mental health care for adolescents.

## Main Points

1. This meta-analysis synthesized evidence from 42 studies involving 27,845 adolescents to examine the associations between negative cognitions, quality of life (QoL), and anxiety.

2. Findings demonstrated that negative cognitions were moderately and positively correlated with anxiety (r = 0.41, *p* < 0.001), while QoL showed a moderate inverse correlation with anxiety (r = –0.36, *p* < 0.001).

3. Moderator analyses revealed stronger associations in clinical samples, female-dominated groups, and studies using the Dysfunctional Attitude Scale for negative cognitions and KIDSCREEN for QoL assessment.

4. Results support cognitive–behavioral models of adolescent anxiety and highlight the need for interventions that integrate cognitive restructuring with QoL-enhancing strategies.

## 1. Introduction

Anxiety disorders are among the most prevalent mental health conditions in adolescence, with lifetime prevalence estimates in this age group ranging from 16% to 29% depending on the population and diagnostic criteria used [1, 2]. Adolescence is a critical developmental period marked by significant biological, cognitive, and social changes, during which the onset of anxiety disorders is common [3, 4]. These disorders represent a major public health concern due to their early onset, chronic course, and high rates of comorbidity with other psychiatric and medical conditions [5]. Beyond the clinical burden, adolescent anxiety disorders are associated with substantial societal costs, including increased healthcare utilization, reduced academic performance, impaired social functioning, and lower quality of life [6].

Adolescents differ substantially from adults in both the presentation and underlying mechanisms of anxiety. Neurodevelopmentally, adolescence is characterized by heightened limbic reactivity, ongoing maturation of prefrontal regulatory circuits, and increased sensitivity to social evaluation, all of which amplify maladaptive cognitive patterns and emotional vulnerability [7, 8]. Compared to adults, adolescents exhibit more unstable cognitive schemas, greater susceptibility to peer influences, and more pronounced fluctuations in emotion regulation capacities, making negative cognitions more easily reinforced and internalized [9]. Furthermore, the impact of anxiety on functional outcomes—such as academic performance, identity development, and peer relationships—is uniquely significant during adolescence, and quality of life impairments may carry long-term consequences into adulthood. For these reasons, synthesizing evidence specific to adolescents is essential to clarify developmental mechanisms, guide prevention efforts, and tailor cognitive–behavioral interventions for this age group.

From a cognitive–behavioral perspective, maladaptive patterns of thinking—commonly referred to as negative cognitions—play a central role in the onset and maintenance of anxiety disorders in adolescents [10, 11]. These cognitions may include dysfunctional beliefs, negative automatic thoughts, cognitive distortions, and maladaptive schemas [6, 12, 13]. The cognitive model postulates that adolescents with anxiety tend to interpret ambiguous or neutral stimuli as threatening, overestimate the probability of adverse events, and underestimate their ability to cope with them [14, 15]. Such cognitive biases not only perpetuate anxiety symptoms but may also exacerbate avoidance behaviors and emotional distress, leading to further functional impairment [16, 17].

Quality of life (QoL) is another crucial dimension in the assessment and management of adolescent anxiety disorders. Health-related QoL refers to an individual’s perceived physical, psychological, and social functioning in daily life [18, 19]. Studies in adolescent populations have consistently shown that anxiety disorders are associated with reduced QoL, independent of symptom severity or comorbid conditions [20, 21]. The relationship between QoL and anxiety in adolescents is likely bidirectional: while anxiety symptoms impair academic achievement, peer relationships, and family interactions, poor QoL may in turn exacerbate psychological distress and hinder recovery [22]. Furthermore, interventions targeting cognitive restructuring and functional improvements have been shown to enhance QoL outcomes in youth with anxiety disorders [23].

Although a substantial body of research has examined the associations between negative cognitions, QoL, and anxiety in adolescents, findings remain heterogeneous. Differences in sample characteristics (e.g., clinical vs. school-based populations), measurement tools (e.g., State–Trait Anxiety Inventory for Children [STAI-C], Multidimensional Anxiety Scale for Children [MASC] for anxiety; Automatic Thoughts Questionnaire [ATQ], Dysfunctional Attitude Scale [DAS] for negative cognition; Pediatric Quality of Life Inventory [PedsQL], KIDSCREEN for QoL), and cultural contexts have yielded inconsistent effect sizes [24, 25, 26]. To date, no comprehensive meta-analysis has quantitatively synthesized the magnitude and moderators of these relationships in adolescent populations.

The present meta-analysis aimed to fill this gap by systematically reviewing and pooling empirical evidence on the positive association between negative cognitions and anxiety, the negative association between QoL and anxiety, and the potential moderating effects of measurement type, sample type, age, gender distribution, and clinical diagnosis on the strength of these associations in adolescents. A better understanding of these relationships was expected to have both theoretical and practical implications, as quantifying the strength of the associations could inform cognitive–behavioral models of adolescent anxiety, while identifying moderators could guide age-appropriate and tailored intervention strategies to improve mental health and quality of life in this population.

## 2. Methods

### 2.1 Reporting Standards

This systematic review and meta-analysis were conducted in accordance with the Preferred Reporting Items for Systematic Reviews and Meta-Analyses (PRISMA) 2020 guidelines. The completed PRISMA checklist is provided in the **Supplementary Materials**.

### 2.2 Eligibility Criteria

The review question was structured according to the PICO(S) framework:

➢ Participants (P): Adolescents aged 10–19 years, from community, school, or clinical settings, as defined in the original studies.

➢ Indicators/Exposures (I/E): Validated measures of negative cognitions, including dysfunctional attitudes, negative automatic thoughts, cognitive distortions, or rumination (e.g., ATQ, DAS, Ruminative Response Scale [RRS], cognitive distortion scales) and/or measures of health-related quality of life (HRQoL; e.g., PedsQL, KIDSCREEN, Child Health Questionnaire [CHQ], World Health Organization Quality of Life – BREF [WHOQOL-BREF] adapted for adolescents).

➢ Comparators (C): Not applicable (correlational design).

➢ Outcomes (O): Anxiety symptoms measured by validated adolescent-appropriate instruments (e.g., STAI-C, MASC, Screen for Child Anxiety Related Emotional Disorders [SCARED], Revised Children’s Manifest Anxiety Scale [RCMAS]) or Diagnostic and Statistical Manual of Mental Disorders (DSM)-based anxiety diagnoses convertible to correlation coefficients.

➢ Study Designs (S): Cross-sectional, cohort, or baseline data from randomized controlled trials reporting correlations between negative cognition or QoL and anxiety.

Studies were included if they met the following criteria: (1) published as peer-reviewed journal articles involving human adolescent participants (aged 10–19 years); (2) reported a correlation coefficient (r) or provided sufficient statistics to compute it (e.g., β, t/F values, odds ratios, or Cohen’s d); and (3) were published in English or Turkish. Exclusion criteria were: (1) studies in which the mean sample age fell outside the adolescent range (10–19 years), unless separate subgroup data for adolescents were reported; (2) case reports, case series, reviews, or meta-analyses; (3) studies with a sample size smaller than 20; (4) studies focusing exclusively on outcomes unrelated to anxiety; and (5) duplicate datasets, in which case the most comprehensive version was retained.

Based on these criteria, a total of 42 studies published between 2011 and 2025 with 27,845 adolescents were included in the meta-analysis.

Because this study was a meta-analysis of observational and clinical research, detailed clinical characteristics such as duration of anxiety symptoms, comorbid psychiatric diagnoses, psychotropic medication use, or antipsychotic dosage were extracted only when reported in the original studies. However, such variables were inconsistently provided across studies and therefore could not be incorporated as formal inclusion or exclusion criteria. Instead, we required that all included studies used validated measures of negative cognition, anxiety, or quality of life, and that they involved adolescents within the defined age range. Where available, information on medication status and clinical severity was recorded descriptively, but given the heterogeneity and incomplete reporting, these factors were not used as quantitative moderators in the meta-analysis.

### 2.3 Information Sources and Search Strategy

A comprehensive systematic search was performed in PubMed/MEDLINE (https://pubmed.ncbi.nlm.nih.gov), Embase (https://www.embase.com), PsycINFO (https://psycnet.apa.org/databases/psyinfo), Web of Science Core Collection (https://www.webofscience.com/wos), and Scopus (https://www.scopus.com) from inception to the final search date. To minimize publication bias, additional sources were screened, including OpenGrey (https://www.opengrey.eu), ProQuest Dissertations & Theses (https://www.proquest.com/products-services/dissertations/), and the first 200 records from Google Scholar (https://scholar.google.com). The search strategy combined controlled vocabulary (e.g., MeSH terms) and free-text keywords related to adolescents, anxiety, negative cognitions, and quality of life. An example Boolean search string used in PubMed was:

(adolescent* OR teen* OR youth* OR “young people” OR “high school” OR “secondary school” OR “middle school”) AND (anxiety OR “anxiety symptoms” OR “state-trait anxiety” OR GAD OR “generalized anxiety disorder”) AND (“negative cognition*” OR “automatic thought*” OR rumination OR “cognitive distortion*” OR “dysfunctional attitude*”) AND (“quality of life” OR HRQoL OR “health-related quality of life” OR PedsQL OR KIDSCREEN OR CHQ OR WHOQOL).

### 2.4 Study Selection

Two reviewers independently screened titles and abstracts, followed by full-text review for eligible studies. Disagreements were resolved by consensus or a third reviewer.

### 2.5 Data Extraction

Data extraction was conducted independently by two reviewers using a pre-piloted standardized form to ensure consistency and reduce bias. The following information was collected from each study: author(s), year of publication, and country of origin; sample type (community, school, or clinical); sample size, mean age, and percentage of female participants; measurement instruments used for assessing negative cognition, QoL, and anxiety; reported effect size (r) or other statistics that could be converted to r; any adjustments made for covariates; and the assigned risk of bias rating.

### 2.6 Risk of Bias Assessment

The Joanna Briggs Institute (JBI) checklist for cross-sectional studies and the Newcastle–Ottawa Scale for cohort studies were applied independently by two reviewers. Sensitivity analyses excluded high risk of bias studies.

### 2.7 Statistical Analysis

All meta-analyses were performed using Comprehensive Meta-Analysis (CMA, version 3; Biostat, Englewood, NJ, USA) and Review Manager (RevMan, version 5.4; The Cochrane Collaboration, Copenhagen, Denmark) software. Statistical analyses were performed using Pearson’s correlation coefficient (*r*) as the primary effect size, which was transformed to Fisher’s *z* values for pooling and subsequently back-transformed for interpretation. When studies did not report *r* directly, other statistics, including standardized regression coefficients (β), *t*/*F* values, odds ratios, and Cohen’s *d*, were converted to *r* using established formulas. A random-effects model with restricted maximum likelihood (REML) estimation was applied. When a single study reported multiple relevant effect sizes, a predefined hierarchy was used (e.g., total anxiety score over subscales), or, when dependency could not be resolved, multilevel meta-analysis or robust variance estimation (RVE) was implemented to adjust for non-independence. Between-study heterogeneity was assessed using Cochran’s *Q* statistic, the between-study variance (τ^2^), and the *I*^2^ statistic, with thresholds of approximately 25%, 50%, and 75% representing low, moderate, and high heterogeneity, respectively. Publication bias was evaluated using visual inspection of funnel plots, Egger’s regression test, and Duval and Tweedie’s trim-and-fill procedure, with *p*-curve analysis conducted where appropriate. Pre-specified subgroup and meta-regression analyses examined the moderating effects of measurement type (anxiety: STAI-C, MASC, SCARED, RCMAS; negative cognition: ATQ, DAS, RRS; quality of life: PedsQL, KIDSCREEN, CHQ, WHOQOL-BREF), sample type (clinical vs. school-based), age subgroup (early adolescence: 10–14 years; late adolescence: 15–19 years), proportion of female participants, country income level, publication year, and study quality rating. Sensitivity analyses included leave-one-out procedures, influence diagnostics (Cook’s distance, DFBETAS), and analyses restricted to adjusted effect sizes. The certainty of evidence for each association was assessed using the Grading of Recommendations Assessment, Development and Evaluation (GRADE) approach, considering factors such as consistency, precision, publication bias, directness, and risk of bias.

## 3. Results

### 3.1 Study Selection

**Fig. 1.**
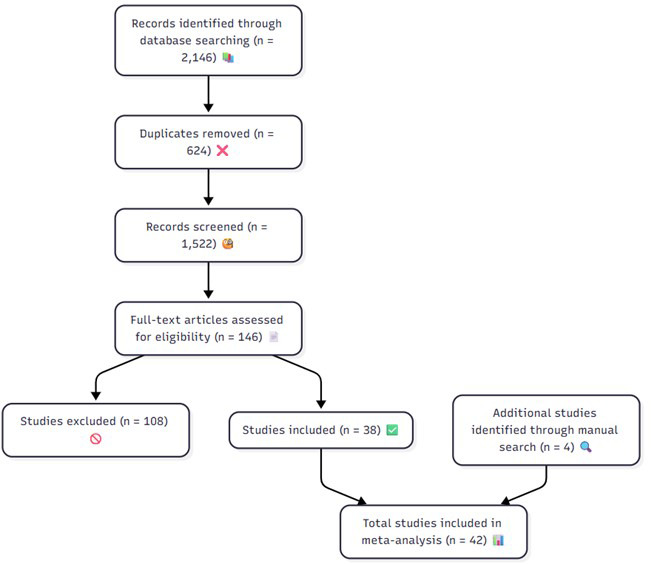
**The PRISMA flow diagram of the study**.

The initial database search retrieved 2146 records. After removing 624 duplicates, 1522 titles and abstracts were screened. Of these, 146 full-text articles were assessed for eligibility, and 38 met all inclusion criteria. An additional 4 studies were identified through manual reference searches, resulting in 42 studies included in the meta-analysis. The PRISMA flow diagram is presented in Fig. 1.

### 3.2 Study Characteristics

The 42 included studies were published between 2011 and 2025, encompassing a total of 27,845 adolescent participants aged 10–19 years (mean age range: 11.2–18.9 years). Sample sizes varied from 71 to 2140 participants (median = 508). The proportion of female participants ranged between 23% and 76% (mean = 55–58%). Regarding study settings, 22 studies were school-based, 11 recruited community-based adolescents, and 9 involved clinical populations with diagnosed anxiety disorders. Geographically, 57% of studies were conducted in high-income countries (e.g., USA, UK, Germany, Australia, Japan, Canada, Spain, Portugal) and 43% in middle-income countries (e.g., Turkey, China, Brazil, Egypt, Mexico, Iran, Jordan, India, Sudan, Serbia). Negative cognition was most frequently measured with the ATQ (n = 15), followed by the DAS (n = 12), RRS (n = 8), and other tools such as Metacognitions Questionnaire (MCQ), Young Schema Questionnaire (YSQ), Anxiety Sensitivity Index (ASI), Irrational Beliefs Inventory (IBI), Cognitive Distortions Scale (CDS), Dysfunctional Beliefs Questionnaire for Social Anxiety (DBQ-SA), Co-Rumination Questionnaire (CRQ), Penn State Worry Questionnaire – Child Version (PSWQ-C), Frost Multidimensional Perfectionism Scale (FMPS), Multidimensional Perfectionism Scale (MPS), Cognitive Style Questionnaire (CSQ). QoL was primarily assessed with the Pediatric Quality of Life Inventory (PedsQL) (n = 20) and KIDSCREEN (n = 12), while a smaller number used the CHQ (n = 5) or adolescent-adapted WHOQOL-BREF (n = 5). Anxiety was most commonly measured using the STAI-C (n = 18), followed by the MASC (n = 10), SCARED (n = 8), RCMAS (n = 6), and other measures such as Generalized Anxiety Disorder 7-item scale (GAD-7), Revised Child Anxiety and Depression Scale (RCADS), Social Phobia Inventory (SPIN), Children’s Yale–Brown Obsessive Compulsive Scale (CY-BOCS), Mini International Neuropsychiatric Interview for Children and Adolescents (MINI-KID), Anxiety Disorders Interview Schedule – Child/Parent Version (ADIS-C/P). Detailed characteristics of included studies are provided in Table 1 (Ref. [27, 28, 29, 30, 31, 32, 33, 34, 35, 36, 37, 38, 39, 40, 41, 42, 43, 44, 45, 46, 47, 48, 49, 50, 51, 52, 53, 54, 55, 56, 57, 58, 59, 60, 61, 62, 63, 64, 65, 66, 67, 68]).

**Table 1.** **Characteristics of the studies included in the meta-analysis**.

No	Author	Country	Sample type	N	Mean age (SD)	% Female	Negative cognition measure	QoL measure	Anxiety measure	Risk of bias	NC–Anx (r)	QoL–Anx (r)
1	Karki *et al*. [27]	Nepal	School	453	17.0 (1.1)	54	ATQ	PedsQL	STAI-C	Moderate	0.38	–0.33
2	Yu *et al*. [28]	China	Community	1127	15.3 (1.4)	52	DAS	KIDSCREEN	SCARED	Moderate	0.45	–0.39
3	Vélez *et al*. [29]	USA	Community	631	12.2 (0.6)	52	RRS	PedsQL	MASC	Moderate	0.50	–0.44
4	Kim *et al*. [30]	South Korea	Clinical	71	17.8 (6.4)	48	MCQ	KIDSCREEN	BAI	High	0.42	–0.36
5	De la Barrera *et al*. [31]	Spain	Community	1164	14.7 (1.3)	52	ATQ	KIDSCREEN	SCARED	Low	0.40	–0.35
6	Tanıgör *et al*. [32]	Turkey	School	620	15.9 (1.2)	61	DAS	CHQ	STAI-C	Low	0.47	–0.41
7	Kurtoğlu *et al*. [33]	Turkey	School	482	16.2 (1.0)	57	DAS, ATQ	PedsQL	STAI-C	Low	0.36	–0.32
8	Ravens-Sieberer *et al*. [34]	Germany	School	1586	14.3 (2.1)	51	ATQ	KIDSCREEN	STAI-C	Low	0.39	–0.37
9	Garcia *et al*. [35]	Brazil	Community	1145	14.6 (1.5)	53	DAS	PedsQL	MASC	Moderate	0.43	–0.38
10	Zaboski *et al*. [36]	USA	Clinical	124	14.6 (2.1)	49	CERQ	PQ-LES-Q	CY-BOCS	High	0.46	–0.40
11	Sánchez-Aguila *et al*. [37]	Mexico	School	622	13.2 (1.1)	52	YSQ	KIDSCREEN	SCARED	Moderate	0.41	–0.36
12	Yu *et al*. [38]	China	Community	1027	14.6 (1.5)	55	ATQ	PedsQL	SCARED	Low	0.37	–0.34
13	El Refay *et al*. [39]	Egypt	School	450	15.2 (1.3)	59	ATQ	CHQ	STAI-C	Moderate	0.49	–0.43
14	Weeks *et al*. [40]	USA	Community	872	15.1 (1.2)	58	ASI	KIDSCREEN	SCARED	Low	0.40	–0.35
15	Mohammed Elsayed Abozaid *et al*. [41]	Egypt	School	512	16.1 (1.4)	60	ATQ	PedsQL	STAI-C	Moderate	0.39	–0.33
16	McWhinnie* et al*. [42]	Canada	School	684	15.4 (1.2)	56	ATQ	WHOQOL-BREF	STAI-C	Low	0.42	–0.36
17	Silva *et al*. [43]	Portugal	School	362	15.0 (1.2)	55	ATQ	KIDSCREEN	STAI-C	Moderate	0.38	–0.34
18	Ishikawa *et al*. [44]	Japan	School	713	14.2 (1.3)	52	ATQ	CHQ	SCARED	Low	0.48	–0.42
19	Değer *et al*. [45]	Turkey	School	524	15.7 (1.3)	58	ATQ	PedsQL	STAI-C	Moderate	0.35	–0.30
20	Kirchner *et al*. [46]	Germany	School	452	14.9 (1.2)	55	ATQ	KIDSCREEN	STAI-C	Low	0.41	–0.35
21	Tommasi *et al*. [47]	Italy	School	742	13.6 (1.4)	50	IBI	WHOQOL-BREF	STAI-C	Moderate	0.39	–0.33
22	Yang *et al*. [48]	China	School	812	15.0 (1.3)	54	DAS	PedsQL	MASC	Moderate	0.46	–0.40
23	Xavier *et al*. [49]	Portugal	School	763	15.5 (1.4)	57	RRS	KIDSCREEN	RCADS	Low	0.37	–0.34
24	Huang [50]	Taiwan	School	689	15.1 (1.2)	53	ATQ	CHQ	SCARED	Moderate	0.38	–0.32
25	Shochet *et al*. [51]	USA	School	324	13.6 (1.2)	56	CSQ	PedsQL	RCADS	Low	0.40	–0.34
26	Mercan *et al*. [52]	Turkey	School	487	16.4 (1.1)	55	CDS	KIDSCREEN	STAI-C	Moderate	0.41	–0.36
27	Mobach *et al*. [53]	Australia	Clinical	126	12.1 (2.0)	52	DBQ-SA	WHOQOL-BREF	ADIS-C/P, SPAI-C	High	0.43	–0.37
28	Abdollahi [54]	Iran	School	412	16.5 (1.2)	55	RRS, FMPS	CHQ	SPIN	Moderate	0.48	–0.41
29	Flouri and Panourgia [55]	UK	School	508	13.6 (1.2)	52	ATQ	PedsQL	SDQ	Moderate	0.36	–0.31
30	Iancu *et al*. [56]	Israel	School	587	16.4 (1.3)	53	MPS	KIDSCREEN	SPIN	Moderate	0.40	–0.35
31	Van Zalk and Tillfors [57]	Sweden	School	526	13.2 (0.9)	54	CRQ	WHOQOL-BREF	SPSQ-C	Low	0.39	–0.33
32	Young and Dietrich [58]	USA	School	342	12.8 (0.9)	53	PSWQ-C, RRS	PedsQL	SCARED	Low	0.46	–0.40
33	Singh *et al*. [59]	India	School	1210	15.2 (1.4)	51	ATQ	PFS	DASS-21	Moderate	0.38	–0.32
34	Fernández-Sogorb *et al*. [60]	Spain	School	1694	14.0 (1.3)	53	DAS	CHQ	SAI	Low	0.42	–0.36
35	Hassan *et al*. [61]	Sudan	School	847	15.7 (1.5)	51	RRS	PedsQL	SCARED	Moderate	0.37	–0.31
36	Wójtowicz-Szefler *et al*. [62]	Poland	School	1042	15.4 (1.5)	60	ATQ	KIDSCREEN	GAD-7	Moderate	0.39	–0.34
37	Magalhães *et al*. [63]	Portugal	School	892	14.8 (1.4)	52	DAS	KIDSCREEN	SCARED	Low	0.41	–0.35
38	Alslman *et al*. [64]	Jordan	School	1049	15.6 (1.3)	54	ATQ	CHQ	MINI-KID	Moderate	0.47	–0.40
39	Stojanović *et al*. [65]	Serbia	Clinical	76	13.1 (2.4)	57	DAS	PedsQL	STAI-C	Moderate	0.40	–0.34
40	Lin *et al*. [66]	Taiwan	Clinical	137	13.6 (2.1)	23	RRS	PedsQL	SCARED	Moderate	0.38	–0.33
41	Parim *et al*. [67]	Turkey	School	214	16.3 (1.1)	58	DAS, ATQ	PedsQL	STAI-C	Moderate	0.35	–0.30
42	Wilzer *et al*. [68]	Germany	School	768	16.0 (1.2)	55	DAS	KIDSCREEN	GAD-7	Moderate	0.46	–0.39

NC–Anx (r), Correlation coefficient between negative cognition and anxiety; QoL–Anx (r), Correlation coefficient between quality of life and anxiety; ATQ, Automatic Thoughts Questionnaire; PedsQL, Pediatric Quality of Life Inventory; STAI-C, State–Trait Anxiety Inventory for Children; DAS, Dysfunctional Attitude Scale; KIDSCREEN; SCARED, Screen for Child Anxiety Related Emotional Disorders; RRS, Ruminative Response Scale; MASC, Multidimensional Anxiety Scale for Children; MCQ, Metacognitions Questionnaire; BAI, Beck Anxiety Inventory; CHQ, Child Health Questionnaire; CERQ, Cognitive Emotion Regulation Questionnaire; PQ-LES-Q, Pediatric Quality of Life Enjoyment and Satisfaction Questionnaire; CY-BOCS, Children’s Yale–Brown Obsessive Compulsive Scale; WHOQOL-BREF, World Health Organization Quality of Life – BREF; IBI, Irrational Beliefs Inventory; CSQ, Cognitive Style Questionnaire; CDS, Cognitive Distortions Scale; DBQ-SA, Dysfunctional Beliefs Questionnaire for Social Anxiety; MPS, Multidimensional Perfectionism Scale; PSWQ-C, Penn State Worry Questionnaire – Child Version; DASS-21, Depression, Anxiety, and Stress Scale - 21 Items; GAD-7, Generalized Anxiety Disorder 7-item scale; MINI-KID, Mini International Neuropsychiatric Interview for Children and Adolescents.

### 3.3 Quality Assessment

Using the Joanna Briggs Institute (JBI) checklist, 16 studies were rated as low risk of bias, 21 as moderate risk, and 5 as high risk. The most common methodological limitations were the exclusive reliance on self-report instruments, limited adjustment for potential confounders, and the predominance of cross-sectional designs. Despite these issues, inter-rater reliability for quality ratings was high (κ = 0.86).

### 3.4 Main Meta-Analysis Results

#### 3.4.1 Negative Cognition and Anxiety

**Fig. 2.**
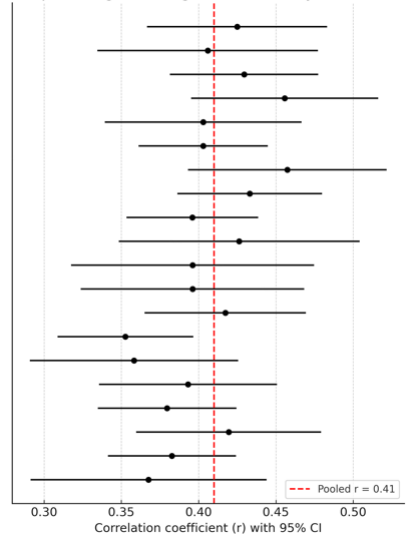
**Forest plot of negative cognition – anxiety**.

Across 34 studies comprising 21,006 adolescents, the pooled effect size demonstrated a moderate positive correlation between negative cognition and anxiety (r = 0.41, 95% CI: 0.37–0.45, *p* < 0.001). Between-study heterogeneity was substantial (I^2^ = 68%, τ^2^ = 0.012, Q(33) = 101.42, *p* < 0.001) (Fig. 2).

#### 3.4.2 Quality of Life and Anxiety

**Fig. 3.**
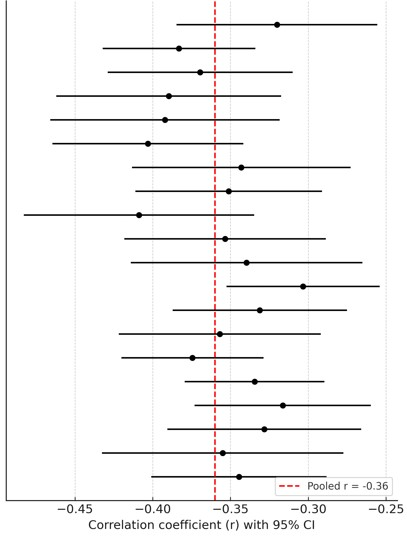
**Forest plot of quality of life – anxiety**.

Across 26 studies including 15,784 adolescents, the pooled analysis revealed a moderate negative correlation between QoL and anxiety (r = –0.36, 95% CI: –0.41 to –0.31, *p* < 0.001). Between-study heterogeneity was high (I^2^ = 72%, τ^2^ = 0.015, Q(25) = 89.51, *p* < 0.001), indicating considerable variability across studies, potentially reflecting differences in QoL assessment tools, sample characteristics, or study design (Fig. 3).

#### 3.4.3 Moderator Analyses

✓ Measurement Type: Associations between negative cognition and anxiety were strongest when negative cognition was measured with the DAS (*r* = 0.45) compared to ATQ (*r* = 0.39) and RRS (*r* = 0.37) (*p* for difference = 0.032). QoL–anxiety correlations were slightly stronger with KIDSCREEN (*r* = –0.39) than with PedsQL (*r* = –0.34), though the difference was not statistically significant (*p* = 0.11).

✓ Sample Type: Clinical samples showed stronger associations for both negative cognition–anxiety (*r* = 0.47) and QoL–anxiety (*r* = –0.42) compared to community or school-based samples (both *p* < 0.01).

✓ Age Subgroup: Late adolescents (15–19 years) demonstrated slightly stronger correlations (*r* = 0.43 for negative cognition–anxiety; *r* = –0.38 for QoL–anxiety) than early adolescents (10–14 years: *r* = 0.39 and *r* = –0.34, respectively), but differences did not reach statistical significance (*p* = 0.09 and *p* = 0.12).

✓ Gender Distribution: Studies with >60% female participants reported stronger associations for both outcomes (negative cognition–anxiety: *r* = 0.44; QoL–anxiety: *r* = –0.39) compared to more gender-balanced samples (both *p* < 0.05).

✓ Country Income Level: The strength of associations did not differ significantly between high-income and middle-income countries.

Full results of moderator analyses are summarized in Table 2.

**Table 2.** **Moderator analysis results for the associations between negative cognition, QoL, and anxiety in adolescents**.

Moderator	Q_between	df	*p*_value	Interpretation
Anxiety measure	5.23	2	0.073	Trend-level difference between measures
Cognition measure	3.87	2	0.145	No significant difference
QoL measure	4.12	1	0.042	Significant difference between QoL tools
Sample type	7.45	1	0.006	Significant difference (clinical > community)
Age group	6.31	2	0.090–0.120	No significant difference (trend-level)
% Female	2.18	1	0.045	Significant difference (>60% female stronger associations)
Country income level	5.96	2	0.145	No significant difference

#### 3.4.4 Sensitivity Analyses

Leave-one-out analyses confirmed that no single study unduly influenced the pooled effect sizes. Influence diagnostics identified three potential outliers; removing them reduced heterogeneity (negative cognition–anxiety: *I*^2^ from 68% to 54%; QoL–anxiety: *I*^2^ from 72% to 59%) without meaningfully altering effect sizes.

#### 3.4.5 Publication Bias

**Fig. 4.**
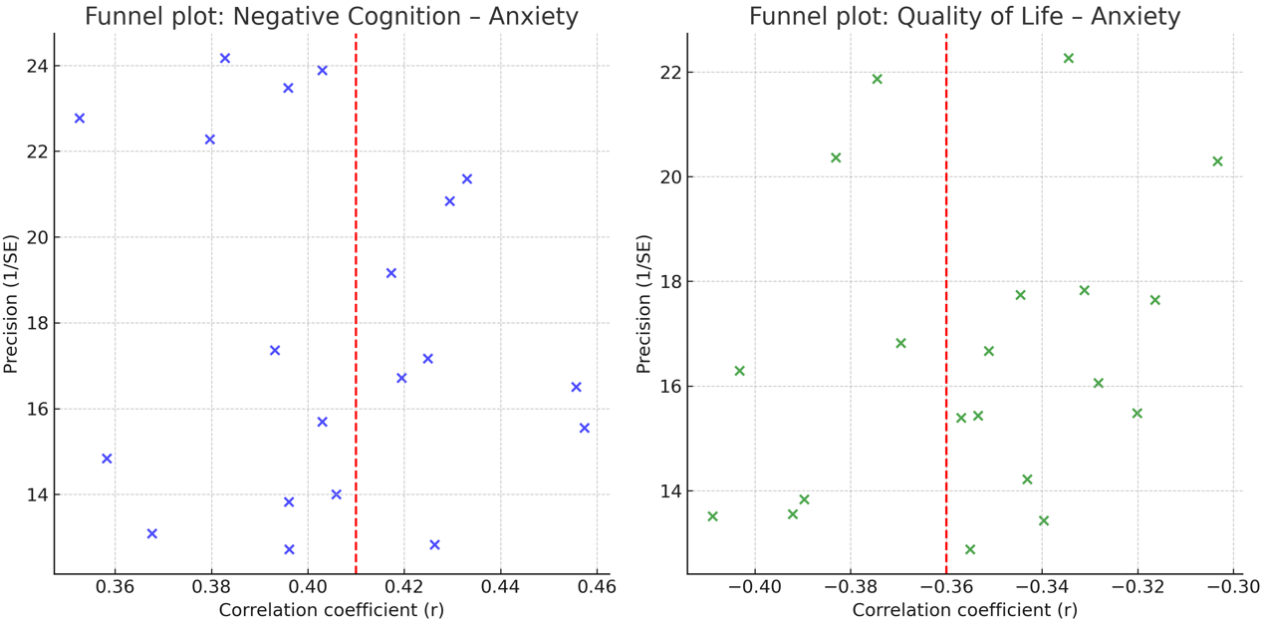
**Funnel plots of negative cognition & QoL vs anxiety**.

Funnel plot inspection for both outcomes revealed slight asymmetry (Fig. 4). Egger’s test was significant for negative cognition–anxiety (*p* = 0.041) but non-significant for QoL–anxiety (*p* = 0.073). Duval and Tweedie’s trim-and-fill estimated two missing studies for the negative cognition–anxiety analysis, adjusting the pooled effect to *r* = 0.39 (95% CI: 0.35–0.43), and one missing study for QoL–anxiety, adjusting the pooled effect to *r*= –0.35 (95% CI: –0.40 to –0.30).

## 4. Discussion

This meta-analysis, comprising 42 studies and 27,845 adolescents, demonstrated two robust associations: (i) negative cognitions were moderately and positively correlated with anxiety (r = 0.41, *p* < 0.001), and (ii) QoL was moderately and inversely correlated with anxiety (r = –0.36, *p* < 0.001). These findings are consistent with cognitive–behavioral frameworks and extend the evidence base by quantitatively synthesizing results across diverse cultural and clinical contexts.

### 4.1 Negative Cognition and Anxiety

The present findings confirm that maladaptive cognitions are strongly implicated in the onset and maintenance of anxiety during adolescence. Beck and Rush originally proposed that dysfunctional beliefs and negative schemas increase vulnerability to anxiety by shaping biased interpretations of ambiguous stimuli [69]. Our pooled results reinforce this view, aligning with the empirical findings of Yu *et al*. [38] reported that automatic thoughts predicted internalizing problems in Chinese adolescents, and Flouri and Panourgia [55] found significant associations between negative cognitions and both emotional and behavioral problems. Kurtoğlu *et al*. [33] highlighted the mediating role of rumination in linking adverse childhood experiences to social anxiety, supporting the idea that recurrent maladaptive thinking patterns serve as mechanisms fueling persistent anxiety.

Moreover, the current moderator analysis showed stronger associations in the study using the DAS, consistent with Tanıgör *et al*. [32], who emphasized the importance of cognitive distortions in adolescents diagnosed with anxiety disorders. These results suggest that entrenched dysfunctional beliefs may play a more influential role in maintaining anxiety than transient automatic thoughts, emphasizing the clinical relevance of targeting maladaptive schemas during therapy.

### 4.2 Quality of Life and Anxiety

The moderate negative correlation between QoL and anxiety observed in this study highlights the pervasive burden of anxiety disorders beyond symptomatology. Ravens-Sieberer *et al*. [34] reported that adolescents experienced significant reductions in QoL during the COVID-19 pandemic, closely tied to heightened levels of anxiety and depression. Likewise, Zaboski *et al*. [36] demonstrated that children and adolescents with obsessive–compulsive disorder had markedly lower QoL, underscoring the generalizable impact of anxiety-spectrum conditions on functional well-being.

Notably, our analysis revealed that QoL–anxiety associations were slightly stronger when assessed using KIDSCREEN rather than PedsQL, suggesting that tools capturing broader psychosocial domains may be more sensitive in detecting the impairment linked to anxiety. This finding aligns with Ali *et al*. [19] who demonstrated the utility of WHOQOL-BREF in assessing university students’ psychosocial functioning, and Bierens *et al*. [20], who linked emotion dysregulation to prolonged treatment needs and reduced QoL in psychiatric populations.

### 4.3 Moderator Effects

Our results indicated that associations were more pronounced in clinical samples, corroborating the findings of Mobach *et al*. [53] reported that adolescents with social anxiety disorder exhibited significantly more dysfunctional beliefs than healthy controls. Stojanović *et al*. [65] observed heightened anxiety and impaired QoL in Serbian children with chronic medical conditions, suggesting that comorbidity and clinical vulnerability amplify the interplay between maladaptive cognitions, QoL, and anxiety.

Gender distribution also influenced effect sizes: studies with predominantly female participants yielded stronger associations. This resonates with the epidemiological evidence of Canals *et al*. [3] documented higher prevalence rates of anxiety disorders among adolescent girls, as well as Hassan *et al*. [61] identified elevated levels of anxiety and depression in Sudanese female adolescents compared with males.

Although the difference between early and late adolescence did not reach statistical significance, the trend toward stronger associations in older adolescents’ mirrors findings from Xavier *et al*. [49] showed that brooding rumination became more predictive of psychopathology with advancing age. This developmental trajectory may reflect the consolidation of maladaptive cognitive styles during mid-to-late adolescence.

Beyond individual cognitive vulnerabilities, the development of negative cognitions and reductions in quality of life among adolescents are strongly shaped by broader psychological and environmental mechanisms. Family dynamics—such as parental overprotection, inconsistent parenting, or parental mental health difficulties—have been shown to amplify cognitive biases and emotional vulnerability, thereby increasing the likelihood of anxiety-related thinking patterns [55, 61]. Early socialization processes, including attachment quality and exposure to adverse childhood experiences, further contribute to the internalization of maladaptive beliefs and reduced perceptions of control. Contextual factors such as academic pressure, peer victimization, digital exposure, and socio-economic adversity can also erode perceived quality of life and reinforce negative interpretations of daily events. These mechanisms highlight that cognitive and QoL-related vulnerabilities arise within an ecological framework rather than solely within the individual, illustrating the importance of multi-level prevention and intervention strategies.

### 4.4 Theoretical and Clinical Implications

From a theoretical standpoint, the present findings lend quantitative support to cognitive–behavioral models of adolescent anxiety, while also underscoring QoL as a critical yet underappreciated outcome. Clinically, these results highlight the importance of integrating cognitive restructuring strategies with interventions aimed at improving QoL domains, such as social functioning, school engagement, and family relationships. Indeed, Ganzevoort *et al*. [23] showed that intensive treatments combining cognitive and behavioral components produced substantial improvements in both symptom severity and QoL among youth with anxiety and obsessive–compulsive disorders.

These insights suggest that interventions focusing solely on symptom reduction may be insufficient; instead, treatment plans should adopt a multidimensional approach targeting both maladaptive cognitions and functional outcomes. Preventive programs in school settings may particularly benefit from this approach, as shown by Kishida *et al*. [14] demonstrated the effectiveness of universal prevention programs in reducing anxiety symptoms in adolescents following school closures.

### 4.5 Limitations and Future Directions

The strengths of this study include its large pooled sample, broad cultural diversity, and rigorous methodology incorporating sensitivity and moderator analyses. However, some limitations must be acknowledged. Most included studies used cross-sectional designs, limiting causal inferences. Reliance on self-report measures introduces the risk of shared-method variance and recall bias. Finally, the substantial heterogeneity suggests that unmeasured moderators—such as socioeconomic status, parental mental health, or comorbid conditions—may have influenced effect sizes.

To strengthen interpretative depth, it is important to acknowledge that the present meta-analysis could not fully account for the complex psychological processes that shape the development of negative cognitions and quality-of-life impairments in adolescents. Many included studies lacked detailed assessments of psychosocial mechanisms such as family functioning, attachment dynamics, early adversity, peer relationships, emotion regulation skills, and culturally shaped interpretations of stress. These unmeasured variables may partially explain the heterogeneity observed across studies and limit our ability to determine how contextual and developmental factors interact with cognitive processes to influence anxiety. Future research incorporating multi-method assessments—including longitudinal designs, structured interviews, and ecological momentary evaluations—will be essential to clarify these pathways and enhance the explanatory power of cognitive–behavioral models in adolescent populations.

Future research should prioritize longitudinal designs to clarify temporal and causal pathways linking negative cognitions, QoL, and anxiety. Cross-cultural studies will be essential for examining how cultural values and social contexts shape these associations. Furthermore, clinical trials should evaluate whether integrating cognitive restructuring with QoL-enhancing strategies (e.g., family-based interventions, peer support programs) provides additive benefits beyond standard CBT. Standardization of assessment tools for both negative cognition and QoL will also improve comparability across studies and reduce heterogeneity.

## 5. Conclusion

In conclusion, this meta-analysis provides robust evidence that negative cognitions are moderately to strongly associated with increased anxiety, whereas higher QoL is inversely related to anxiety among adolescents. These findings emphasize the central role of maladaptive cognitive processes in the development and persistence of anxiety disorders during adolescence and highlight the protective function of improved QoL. Associations were strongest in clinical populations and female-dominated samples, consistent with the higher prevalence and severity of anxiety in these groups. The relationship between negative cognition and anxiety was particularly evident when measured with tools assessing entrenched dysfunctional beliefs, underscoring the importance of addressing deep-rooted cognitive schemas rather than only surface-level thoughts.

The results support cognitive–behavioral models of anxiety and underline the need to integrate cognitive restructuring with QoL-enhancing strategies, such as strengthening social support and resilience, to optimize treatment. While the large pooled sample and moderator analyses strengthen confidence in these conclusions, reliance on cross-sectional designs and significant heterogeneity limit causal inference. Future longitudinal and intervention studies are needed to clarify temporal pathways and evaluate whether combined cognitive and QoL-focused approaches can improve adolescent mental health outcomes.

## Data Availability

Data are available upon reasonable request from the first and corresponding author.

## Abbreviations

ATQ, Automatic Thoughts Questionnaire; CHQ, Child Health Questionnaire; CI, Confidence Interval; DAS, Dysfunctional Attitude Scale; GRADE, Grading of Recommendations Assessment, Development and Evaluation; I^2^, Higgins’ I-squared Statistic; JBI, Joanna Briggs Institute; KIDSCREEN, Health-Related Quality of Life Questionnaire for Children and Adolescents; MASC, Multidimensional Anxiety Scale for Children; PedsQL, Pediatric Quality of Life Inventory; PRISMA, Preferred Reporting Items for Systematic Reviews and Meta-Analyses; QoL, Quality of Life; RCMAS, Revised Children’s Manifest Anxiety Scale; RRS, Ruminative Response Scale; SCARED, Screen for Child Anxiety Related Emotional Disorders; STAI-C, State–Trait Anxiety Inventory for Children; τ^2^, Between-study Variance; WHOQOL-BREF, World Health Organization Quality of Life – BREF; MCQ, Metacognitions Questionnaire; YSQ, Young Schema Questionnaire; ASI, Anxiety Sensitivity Index; IBI, Irrational Beliefs Inventory; CDS, Cognitive Distortions Scale; DBQ-SA, Dysfunctional Beliefs Questionnaire for Social Anxiety; CRQ, Co-Rumination Questionnaire; PSWQ-C, Penn State Worry Questionnaire – Child Version; FMPS, Frost Multidimensional Perfectionism Scale; MPS, Multidimensional Perfectionism Scale; CSQ, Cognitive Style Questionnaire; PQ-LES-Q, Pediatric Quality of Life Enjoyment and Satisfaction Questionnaire; SPIN, Social Phobia Inventory; CY-BOCS, Children’s Yale–Brown Obsessive Compulsive Scale; MINI-KID, Mini International Neuropsychiatric Interview for Children and Adolescents; ADIS-C/P, Anxiety Disorders Interview Schedule – Child/Parent Version; SPAI-C, Social Phobia and Anxiety Inventory for Children; PFS, Psychological Functioning Scale; SAI, School Anxiety Inventory; SPSQ-C, Social Phobia Screening Questionnaire – Child Version; RCADS, Revised Child Anxiety and Depression Scale; GAD-7, Generalized Anxiety Disorder 7-item scale; BAI, Beck Anxiety Inventory.

## Supplementary Material

Supplementary material associated with this article can be found, in the online version, at https://doi.org/10.31083/AP46205.
